# Conservation of Forest Birds: Evidence of a Shifting Baseline in Community Structure

**DOI:** 10.1371/journal.pone.0011938

**Published:** 2010-08-02

**Authors:** Chadwick D. Rittenhouse, Anna M. Pidgeon, Thomas P. Albright, Patrick D. Culbert, Murray K. Clayton, Curtis H. Flather, Chengquan Huang, Jeffrey G. Masek, Susan I. Stewart, Volker C. Radeloff

**Affiliations:** 1 Department of Forest and Wildlife Ecology, University of Wisconsin-Madison, Madison, Wisconsin, United States of America; 2 Department of Statistics, University of Wisconsin-Madison, Madison, Wisconsin, United States of America; 3 United States Department of Agriculture Forest Service, Rocky Mountain Research Station, Fort Collins, Colorado, United States of America; 4 Department of Geography, University of Maryland, College Park, Maryland, United States of America; 5 Biospheric Sciences, NASA Goddard Space Flight Center, Greenbelt, Maryland, United States of America; 6 United States Department of Agriculture Forest Service, Northern Research Station, Evanston, Illinois, United States of America; University of Zurich, Switzerland

## Abstract

**Background:**

Quantifying changes in forest bird diversity is an essential task for developing effective conservation actions. When subtle changes in diversity accumulate over time, annual comparisons may offer an incomplete perspective of changes in diversity. In this case, progressive change, the comparison of changes in diversity from a baseline condition, may offer greater insight because changes in diversity are assessed over longer periods of times. Our objectives were to determine how forest bird diversity has changed over time and whether those changes were associated with forest disturbance.

**Methodology/Principal Findings:**

We used North American Breeding Bird Survey data, a time series of Landsat images classified with respect to land cover change, and mixed-effects models to associate changes in forest bird community structure with forest disturbance, latitude, and longitude in the conterminous United States for the years 1985 to 2006. We document a significant divergence from the baseline structure for all birds of similar migratory habit and nest location, and all forest birds as a group from 1985 to 2006. Unexpectedly, decreases in progressive similarity resulted from small changes in richness (<1 species per route for the 22-year study period) and modest losses in abundance (−28.7–−10.2 individuals per route) that varied by migratory habit and nest location. Forest disturbance increased progressive similarity for Neotropical migrants, permanent residents, ground nesting, and cavity nesting species. We also documented highest progressive similarity in the eastern United States.

**Conclusions/Significance:**

Contemporary forest bird community structure is changing rapidly over a relatively short period of time (e.g., ∼22 years). Forest disturbance and forest regeneration are primary factors associated with contemporary forest bird community structure, longitude and latitude are secondary factors, and forest loss is a tertiary factor. Importantly, these findings suggest some regions of the United States may already fall below the habitat amount threshold where fragmentation effects become important predictors of forest bird community structure.

## Introduction

Armed with biological diversity information, scientists and conservationists frequently ask questions about biological diversity changes in response to habitat loss or fragmentation [1], disturbance and land-use change [2], and climate change [3]. Tenable measures of biological diversity are essential to answering these questions [4]. Biological diversity is commonly defined as species richness (the number of species present in an area) and less often as species diversity or community structure, the number of species weighted by their abundance (e.g., evenness) [5]. Species richness data do not require abundance data, the collection of which requires investments of labor and time. However, the use of species richness as the sole basis for quantifying changes in biological diversity is limited when there is species turnover (change in species composition), a large change in species abundance, or a difference in sampling effort over space and time [6]. In addition, comparisons of changes in biological diversity between samples adjacent in time (i.e., successive change) may mask substantial shifts in diversity that accumulate over time because short-term reference conditions are used to assess change (i.e., the shifting baseline syndrome) [7].

The shifting baseline syndrome arises when environmental conditions degrade over time, yet contemporary observers falsely perceive less change than perceived by historic observers due to their limited time in the system [7]. For example, in the fisheries literature, the shifting baseline syndrome is synonymous with precipitous declines to the point that current communities contain a small fraction of the number of species or species' abundance that was present historically [8]. With respect to community structure, the shifting baseline does not have an inherently negative connotation; it simply means that similarity of communities tends to decrease between two samples as the time between samples increases (i.e., progressive change) [9]. Therefore, measures of *progressive change* in community similarity may provide greater insight than richness or abundance when community structure changes, especially over a long time period.

Changes in avian community structure may occur from successional changes in forest vegetation and from disturbance events [10], [11]. Forest disturbance can take several pathways, 1) forest disturbance that results in forest loss with no change in fragmentation (i.e., conversion of forest to non-forest); 2) forest disturbance that results in forest fragmentation (i.e., conversion of forest to non-forest, with a decrease in forest size and an increase in isolation of remnant forest patches); and 3) forest disturbance that alters vegetation structure and composition while maintaining a forested state. Clearly, forest loss and forest fragmentation contribute greatly to declines in forest avifaunal biodiversity [12]. What is less clear is the role of forest disturbance in avian community structure. In general, one would expect forest disturbance to increase or maintain bird species richness provided the disturbance occurs at a frequency and intensity intermediate with respect to the rate of vegetation recovery from disturbance [13] and without conversion to a non-forested state (forest loss). For example, sustainable levels of tree harvest with maintenance of core areas of mature forest, implemented in Missouri oak-hickory forests, had minimal impact on late-successional forest bird species, a case of intermediate intensity disturbance [14]. Similarly, a 24-year study of group-selection tree harvest in Maine reported a short-term increase in abundance of early successional bird species with little change in abundance of late successional species in adjacent, unharvested forest, a case of intermediate frequency disturbance [15]. However, infrequently disturbed areas or highly disturbed areas may experience sharp declines in richness or abundance following a disturbance event [16].

**Figure 1:**
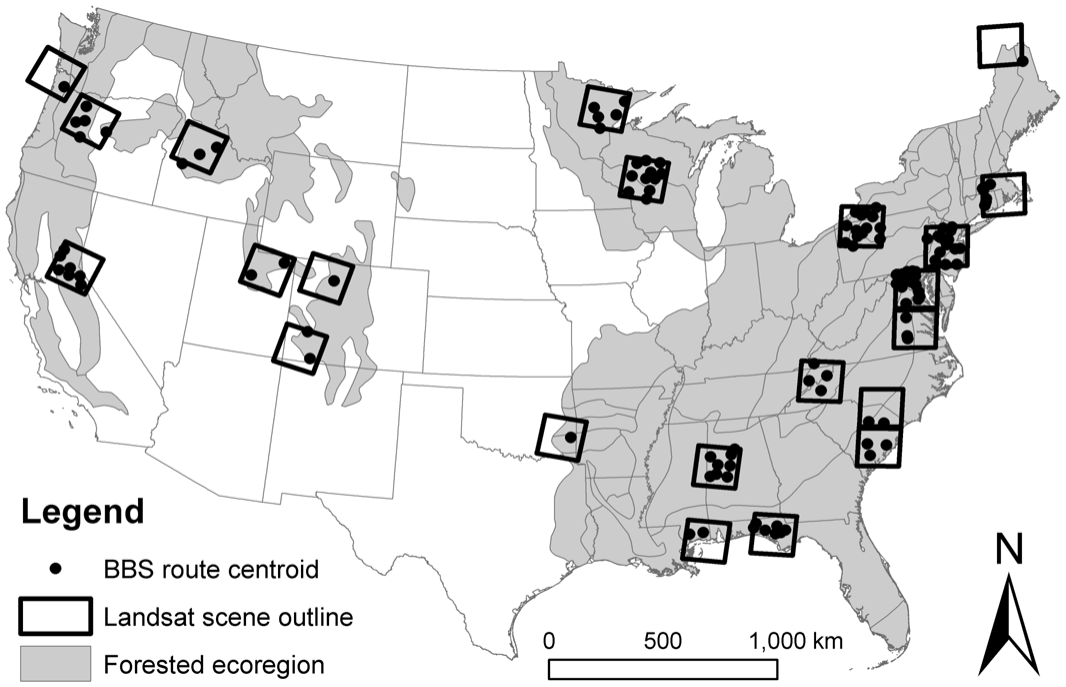
Location of the 122 Breeding Bird Survey (BBS) routes with sufficient data for analysis and corresponding Landsat scene outlines.

Our objectives were to determine how avian community structure has changed in forests of the conterminous United States over a 22-year period and whether those changes were associated with forest disturbance. We used North American Breeding Bird Survey data and a time series of Landsat images classified with respect to land cover change (Figure 1), and mixed-effects models, to accomplish this objective. Given known population declines for many species of birds [17], we expected changes in avian community structure over time. We hypothesized that species which share specific behavioral traits or functional roles respond similarly to forest disturbance. Therefore, we grouped species into guilds based on migratory habit (Neotropical migrants, temperate migrants, or permanent residents) and nest location (ground nesters, mid-story and canopy nesters, cavity nesters, or interior forest nesters [18], [19]; see Table S1 in Supporting Information for scientific names and forest guild memberships). We expected greater changes in Neotropical migrant and temperate migrant guilds than the permanent resident guild because the former may more readily relocate following forest disturbance, whereas the latter tend to have more general habitat requirements (making use of many forest successional states) allowing them to persist in the face of disturbance [20]. We also expected greater changes in the mid-story and canopy and interior nesting guilds than for the ground nesting guild because the former have reduced nest site availability in canopy-removing disturbance events and when interior forest is perforated by disturbance.

## Results

### Changes in avian community structure

**Figure 2:**
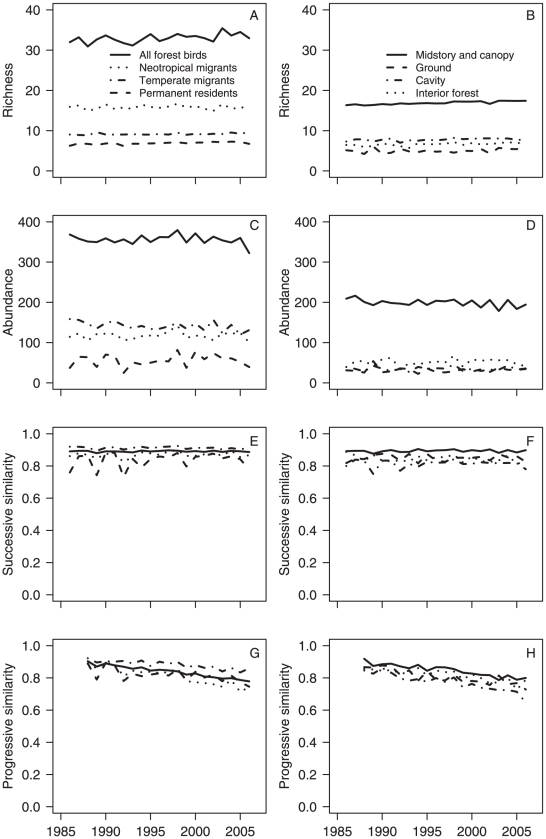
Changes in forest bird community structure by migratory habit (left column) and nest location (right column). Progressive change in community similarity values compares proportional abundance of species for each route and year to the route's baseline forest bird community (1985–1987).

We found evidence of significant but subtle changes in richness over the 22-year study period for Neotropical migrants (annual trend of −0.036×22 years = −0.79 species per route), permanent residents (+0.84 species), and cavity nesters (+0.37 species) (Table 1; Figure 2a,b). As expected, we found significant changes in abundance over the 22-year study period for nearly all guilds examined (Table 1; Figure 2c,d). Abundance of all forest birds as a group decreased by 28.7 individuals. Temperate migrants had the largest decrease in abundance (22.1 individuals) among migratory habit guilds. Cavity nesters (6.6 individuals) and permanent residents (11.2 individuals) had the only significant increases in abundance among all guilds examined. Collectively, these changes in richness and abundance did not produce significant trends in successive similarity (year-to-year changes) for any guild except Neotropical migrants, indicating that successive similarity was constant over time (Table 1; Figure 2e,f). However, we found differences in the inherent successive similarity values among migratory habit and nest location guilds, as indicated by the different intercepts (Figure 2e,f).

**Table 1:** Mean annual trends in forest bird community structure over time for 122 Breeding Bird Survey routes located in the forested ecoregions of the conterminous United States, 1985–2006.

			Community similarity	
Guild	Richness^a^	Abundance	Successive	Progressive
*Migratory habit*				
Neotropical migrants	**−0.036**	**−0.465**	**0.0010**	**−0.0068**
Temperate migrants	−0.003	**−1.006**	0.0006	**−0.0036**
Permanent residents	**0.038**	**0.508**	0.0014	**−0.0030**
*Nest location*				
Ground nesters	−0.011	**−0.409**	−0.00001	**−0.0076**
Mid-story and canopy nesters	0.001	**−1.000**	−0.0005	**−0.0068**
Cavity nesters	**0.017**	**0.299**	−0.0006	**−0.0065**
Interior forest nesters	0.015	0.021	−0.0013	**−0.0089**
*All forest birds*	−0.015	**−1.305**	0.0004	**−0.0053**

a Bold denotes significance at p≤0.05.

When estimating community similarity progressively, a significant pattern of decreasing similarity to the baseline structure over time was evident for all migratory habit and nest location guilds, and all forest birds as a group (Table 1). The 1985–1987 baseline community for all routes combined contained 162 species with an average 3-year abundance of 289 individuals. By 2006, the median community similarity to that baseline had declined to 0.779 for all forest birds (Figure 2g). Among all guilds in 2006, community similarity was lowest for cavity nesters (0.648) and ground nesters (0.728) and highest for temperate migrants (0.860) and mid-story and canopy nesters (0.800) (Figure 2g,h).

Without widespread evidence for significant successive change in forest bird communities, we proceeded with analyses of the effects of forest disturbance using progressive change.

### Effects of forest disturbance on avian community structure

**Figure 3:**
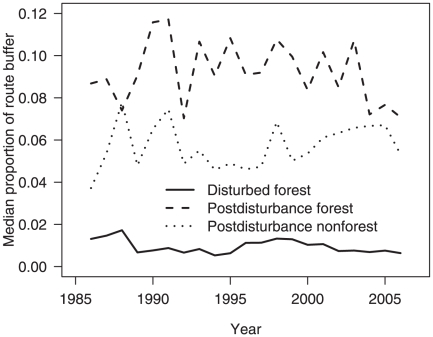
Median proportion of disturbed forest, post-disturbance forest (regenerating forest) and post-disturbance non-forest (forest loss) within a 1200-km^2^ circular landscape surrounding Breeding Bird Survey routes located in forested ecoregions of the conterminous United States. See Fig. 1 for locations of Breeding Bird Survey routes.

Within Breeding Bird Survey route buffers in forested ecoregions of the conterminous United States, the mean proportion of disturbed forest was 0.014 (SE 0.0004, range 0.0001–0.105, n = 1315 route-year observations) and the mean proportion of persistent forest was 0.479 (SE 0.017, range 0.109–0.886, n = 122 routes). We found no significant trend in the median proportion of disturbed forest over time (Spearman-rank test, *r_s_* = −0.330, p-value = 0.144), indicating that the proportion of disturbed forest was relatively constant over time (Figure 3). However, we found evidence that forest disturbance rates and changes in progressive similarity were influenced by initial forest conditions. When ranked by amount of persistent forest in the first year of each routes' respective time series of images, the top 20 routes (range of proportion of persistent forest 0.72–0.89) had lower rates of forest disturbance (Wilcoxon signed-rank test, p-value<0.038) and lower progressive similarity values (Wilcoxon signed-rank test, p-value = 0.020) than the bottom 20 routes (range of proportion of persistent forest 0.11–0.29).

When placed within the spatio-temporal context of the mixed-effects, repeated measures analysis we found support for current and past forest disturbances affecting progressive similarity for nearly all of the forest bird guilds examined (Table 2). Among migratory habit and nest location guilds, disturbed forest was the most-supported model for 2 guilds. When coupled with the outcome of forest disturbance (i.e., regenerating forest or forest loss), disturbed forest was present in most-supported models for 3 additional guilds. Forest loss was present in only 1 of the 8 most-supported models.

**Table 2:** Support (Akaike weights) for models of progressive similarity and forest disturbance, by guild, on Breeding Bird Survey routes located in forested ecoregions of the conterminous United States, 1985–2006.

			Persistent forest		Disturbed forest		Post-disturbance forest		Post-disturbance non-forest		Disturbance and post-disturbance forest		Disturbance and post-disturbance non-forest	
Guild	Full	LL	A	B	A	B	A	B	A	B	A	B	A	B
*Migratory habit*														
Neotropical migrants	0.00	0.00	0.10	0.00	**0.45**	0.00	0.16	0.00	0.16	0.00	0.06	0.00	0.06	0.00
Temperate migrants	0.04	0.00	0.07	**0.88**	0.00	0.00	0.00	0.00	0.00	0.00	0.00	0.00	0.00	0.00
Permanent residents	0.00	0.07	0.00	0.01	0.00	**0.54**	0.00	0.04	0.00	0.02	0.00	0.20	0.00	0.12
*Nest location*														
Ground nesters	0.00	0.01	0.01	0.01	0.07	0.00	0.31	0.00	0.04	0.00	**0.52**	0.00	0.03	0.00
Mid-story or canopy	0.07	0.00	0.04	**0.85**	0.01	0.00	0.00	0.00	0.02	0.00	0.00	0.00	0.00	0.00
Cavity nesters	0.00	0.00	0.00	0.00	0.02	0.00	0.00	0.00	0.37	0.00	0.00	0.00	**0.61**	0.00
Interior forest nesters	0.00	0.00	**0.32**	0.00	0.03	0.00	**0.32**	0.00	0.01	0.00	**0.32**	0.00	0.01	0.00
*All forest species*	0.00	0.00	**0.94**	0.05	0.01	0.00	0.00	0.00	0.00	0.00	0.00	0.00	0.00	0.00

LL = Latitude and Longitude. A is model without Latitude and Longitude, B is model with Latitude and Longitude. Most-supported model within each guild bolded for emphasis.

Unexpectedly, the direction of the effect of forest disturbance on progressive similarity was positive, indicating that forest disturbance maintained or increased similarity to the baseline community for Neotropical migrants, permanent residents, ground nesters, and cavity nesters (Table 3). Additionally, large areas of post-disturbance non-forest and post-disturbance forest increased progressive similarity for cavity nesters and ground nesters, respectively. Progressive similarity for all forest birds as a group, and for temperate migrants, and mid-story and canopy nesters decreased with an increase in the amount of persisting forest (Table 3).

**Table 3:** Parameter estimates, standard errors, and *t* values for attributes of the 1200 km^2^ circular landscape surrounding Breeding Bird Survey routes that affect progressive community similarity of forest birds.

Guild	Estimate	Std. Error	*t* value
Neotropical migrants			
Intercept	0.7791	0.0124	63.07
Year	−0.0068	0.0005	−13.30
Disturbed forest	0.2210	0.2652	0.83
Temperate migrants			
Intercept	0.8482	0.0083	102.46
Year	−0.0036	0.0005	−6.52
Longitude	0.0032	0.0006	5.69
Latitude	0.0055	0.0021	2.67
Persistent forest	−0.2110	0.0442	−4.77
Permanent residents			
Intercept	0.7642	0.0134	56.82
Year	−0.0031	0.0007	−4.44
Longitude	0.0031	0.0009	3.40
Latitude	−0.0180	0.0031	−5.76
Disturbed forest	0.9620	0.3846	2.50
Ground nesters			
Intercept	0.7702	0.0146	52.89
Year	−0.0048	0.0008	−5.75
Longitude	−0.0025	0.0010	−2.58
Latitude	−0.0114	0.0038	−3.00
Disturbed forest	0.4861	0.4517	1.08
Post-disturbance forest	0.2207	0.1446	1.53
Mid-story and canopy nesters			
Intercept	0.8192	0.0088	93.46
Year	−0.0053	0.0005	−10.26
Longitude	0.0031	0.0006	5.33
Latitude	0.0084	0.0022	3.88
Persistent forest	−0.2001	0.4695	−4.26
Cavity nesters			
Intercept	0.7544	0.0113	66.67
Year	−0.0069	0.0008	−8.92
Disturbed forest	0.7639	0.4070	1.88
Post-disturbance non-forest	0.5205	0.1519	3.43
Interior forest nesters^1^			
Intercept	0.7626	0.0156	48.88
Year	−0.0062	0.0008	−7.42
Persistent forest	0.2424	0.0793	3.06
Disturbed forest	−0.5566	0.4442	−1.25
Post-disturbance forest	−0.3967	0.1320	−3.01
All forest species			
Intercept	0.8218	0.0077	106.82
Year	−0.0053	0.0004	−12.39
Persistent forest	−0.1607	0.0377	−4.27

1 Values were averaged across top 3 competing models.

*t* values exceeding 1.96 meet significance threshold considering fixed effects only [53].

In addition to forest disturbance, we found significant effects of latitude and longitude on progressive similarity for many of the guilds examined (Table 3). Moving from South to North, progressive similarity decreased for permanent residents and ground nesters, and increased for temperate migrants and mid-story and canopy nesters. Moving from West to East, progressive similarity increased for temperate migrants, permanent residents, and mid-story and canopy nesters, and decreased for ground nesters.

## Discussion

In contrast to previous studies of shifting baselines utilizing >50 year datasets [8], [21], [22], our results indicate contemporary forest bird community structure is changing rapidly over a relatively short period of time (e.g., ∼22 years). In fragmented forests, long term changes in community similarity arise from turnover in species [23] and from variability in species richness (i.e., local extinctions with variable recolonization) [24]. Our observed changes in community structure occurred despite little change in species richness and were associated with forest disturbance and forest regeneration. These findings suggest that forest disturbance and forest regeneration are primary factors affecting forest bird community structure, with forest loss playing a lesser role.

While progressive similarity decreased over time for all forest species as a group, we found considerable variation in progressive similarity among migratory habit and nest location guilds (Figure 2). Decreases in progressive similarity may be related to declines in richness or abundance relative to the historic baseline, or they may be related to increases in richness or abundance relative to the historic baseline. Thus, it is important to note that a decrease in community similarity may reflect degradation or restoration of a forest community. Not surprisingly we found evidence of changes in forest Neotropical migrant communities, as have other studies [12] and toward which many conservation efforts such as Partners in Flight are targeted. However, we found, unexpectedly, that progressive similarity was as low for permanent residents as for Neotropical migrants. This is in contrast to the literature which indicates that permanent resident populations are relatively robust to changes in landscape structure [25], [26]. A potential mechanism for the effects of forest disturbance on community structure of permanent residents is extinction-colonization dynamics as a function of population size, i.e., common resident species are most likely to exhibit local extinction in small forests where population sizes are small [27]. Coupled with this, resident species are also susceptible to local extinction caused by environmental extremes, including severe winters with heavy snow cover [28], heat waves [29], and drought [30], [31]. Colonization dynamics of resident species may be driven by dispersal capability, inter-patch distances, and the regional species pool [32], all of which may be influenced by fragmentation [33]. Our analysis shows that forest permanent resident communities have changed as rapidly as forest Neotropical migrant communities over the 22-year study period, perhaps because they are exposed to habitat conditions in the local landscape throughout their lifetime. Given this apparent sensitivity to the local landscape, using progressive similarity of permanent residents as a “canary in the coal mine” indicative of the stability of forest conditions that broadly determine habitat conditions for all birds (e.g., disturbance, fragmentation, range expansions, and climate change) may be useful way to monitor trends among the full avian community.

In contrast to our initial expectations, forest disturbance maintained or increased progressive similarity of Neotropical migrants, permanent residents, ground nesters, and cavity nesters. Two lines of evidence may be used to place these findings in an ecological context. First, we found that routes with low persistent forest have higher rates of forest disturbance and higher progressive similarity values than routes with high persistent forest. Forest disturbance has the greatest effects on biodiversity when it results in fragmentation or there is fairly little habitat remaining [34]. While our landscapes remained largely forested (mean proportion of persistent forest = 0.479), routes with low persistent forest (range 0.11–0.29) may already fall below the habitat amount threshold where fragmentation effects become important predictors of species richness and abundance (approximately 10–30% suitable habitat) [34], [35], [36]. Sustained forest disturbance over the 22-year period of our study may maintain habitat similarity and thus, progressive similarity on low persistent forest routes. In contrast, routes with high persistent forest (range 0.72–0.89) may have more specialist forest species (i.e., late successional species, interior forest species) and greater variation in extinction-colonization dynamics of those species following disturbance events and thus, lower progressive similarity values [32].

Second, the intermediate disturbance hypothesis suggests that species richness is highest in landscapes with non-catastrophic disturbance events that occur at moderately frequent return intervals [13]. The Breeding Bird Survey is a roadside survey [37], thus all routes were established in landscapes that, due to initial construction and ongoing maintenance activities, contain roadside habitat held in a relatively constant successional and structural state over the period of the study (Figure 3). Our results suggest that forest disturbance maintains progressive similarity in eastern forests of the United States, provided there is little to no forest loss. However, we did not classify disturbance with respect to source; thus we do not know whether forest disturbance from anthropogenic sources vs. natural sources contributed equally to community similarity.

To our knowledge, we are the first to document spatio-temporal gradients of higher progressive similarity in the eastern than in the western United States. Latitudinal gradients in species diversity, with highest diversity near the equator and declining towards the poles, are well established [38]. However, longitudinal gradients in species diversity are less well known for the conterminous United States. Forest bird population responses to forest fragmentation and landscape change are more difficult to detect in western than in eastern forests [39], [40] due in part to greater inherent variability in nest predation rates and brood parasitism by cowbirds that may mask responses to forest fragmentation in western landscapes [41], [42]. This east-west contrast also arises from differences in matrix habitat – fewer forests in the West have been converted to agriculture and urban than in the East [40] – and in the time elapsed since fragmentation and development occurred in western and eastern landscapes [42]. Additionally, western landscapes have more variable fire history [43] and stronger elevation gradients in moisture and temperature [44] than eastern landscapes. Taken together, these studies and our results suggest mechanisms explaining why western forest bird communities have a stronger response to forest disturbance and forest regeneration, with greater changes in progressive similarity, than eastern forest bird communities.

While our empirical results are specific to forest birds, the pattern of decay in progressive similarity that we identified raises important issues regarding patterns of community diversity change over time for other taxa. Many studies use annual monitoring to quantify changes in richness or abundance of communities, yet few studies explicitly test for progressive changes in community similarity. Both successive and progressive similarity offer valuable information regarding patterns of change in community similarity at different temporal scales. For example, when a discrete instance of change in community structure may be expected, such as following a large, intense disturbance event, successive similarity may reveal a substantial change in community structure [11]. However, successive similarity may offer a false sense of security regarding the status of communities when relatively small changes in richness or abundance values accumulate over time, resulting in substantial long-term changes in communities when compared to historic baseline conditions that successive similarity cannot detect. For birds and other taxa, progressive change over time should be expected and thus a longer time horizon is needed to determine whether these changes warrant concern or signal a problem. In this way, conservation or management efforts may benefit from the use of metrics such as progressive similarity that can quantify change in communities over a long time period and reveal progressive change.

## Methods

### Assessment of changes in avian community structure

We assessed changes in avian community structure for the period 1985–2006 using the North American Breeding Bird Survey (BBS). The BBS is an annual, roadside survey of >4000 routes, each 39.4 km long, in the United States and Canada. Our study included 122 BBS routes (1315 route-year observations) representing nine forested ecoregions within the conterminous United States (Figure 1). Volunteers conducted 50 three-minute point counts at 0.8 km intervals along each route. Data consisted of counts of individual birds seen or heard during each point count, identified to species, and tallied by route. We further grouped route-level richness and abundance data by guild for all analyses.

A concern with studies of communities is the loss of information that occurs when characterizing changes in diversity as a single metric, whether richness, abundance, or community similarity, because of species turnover and changes in species abundance over time. This loss of information is inevitable and a property of any diversity measure. Thus, the choice of diversity measure should depend on how much information is needed to answer the question at hand. In our case, we used the Yue-Clayton index of community similarity [45] because it is sensitive to changes in four common measures of diversity: richness, abundance, composition, and evenness [46]. We determined community similarity for each route by guild as:
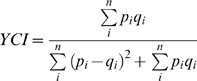
(1)where *p_i_* is the proportional abundance of species *i* in community *p*, *q_i_* is the proportional abundance of species *i* in community *q*, and *n* is the pooled count of species observed in communities *p* and *q* [45]. To quantify community similarity successively (i.e., year-to-year), *p* represented abundance for the community of a route in year *t* and *q* represented abundance for the community of the same route in year *t*+1. To quantify community similarity progressively (i.e., year-to-baseline), we designated *p* as the abundance of the initial community of a route, defined as the average abundance by species for the years 1985–1987, and *q* as the community of the same route in each subsequent year. The Yue-Clayton index ranges from 0 (completely different communities) to 1 (identical communities) and is identical to Jaccard's index when proportional abundance of two populations is uniform [45].

A second concern with studies of communities is the quality of the initial diversity assessment (i.e., baseline) and subsequent annual assessments, typically measured as completeness with respect to the estimated or total species pool for a site. The baseline community captured on average 72% of the total species pool observed across all years (1985–2006) for each site. Had we defined the baseline community as 1985 only, we would have captured on average 61% of the total species pool. Thus, defining the baseline community as a three-year average offered greater completeness than using only the first year. Many factors affect the completeness of a survey, including differences in sampling effort, observers, weather, or detectability of species or individuals. Data screening procedures and sophisticated modeling techniques (see Statistical analyses, below) can minimize or control for these sources of variation in completeness. Thus, by following these procedures and modeling sources of variation, we assumed that changes in similarity that we found were due to changes in bird community structure over time, and not due to incomplete detections.

### Association between forest disturbance and avian community structure

We assessed land cover change for 22 study areas within the conterminous United States from a series of annual or biennial Landsat TM/ETM+ imagery for the period 1985–2006 [47] (Figure 1). Each study area was approximately 185 km×185 km, corresponding to the size of a Landsat image, and contained at least 15% forest cover. For each image in the time series, we quantified the probability of a pixel being a forest pixel using the Integrated Forest Index:
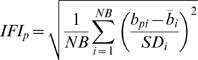
(2)where 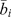 and 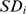 are the mean and standard deviation of forest training pixels within that image for band *i*, 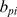 is the band *i* spectral value for pixel *p*, and *NB* is the number of spectral bands. Forest training pixels were defined by a peak (or threshold) in the histogram of reflectance values from TM band 3 (red band) [48]. When calculated over multiple spectral bands (TM/ETM+ bands 2, 3, and 7), the Integrated Forest Index approximates the inverse probability of a pixel being forested as the normalized distance to the center of delineated forest training pixels within that image in the spectral space. Small Integrated Forest Index values indicate strong similarity with the spectral center of the forest training pixels and a high probability of a pixel being forested.

We applied a vegetation change tracker algorithm to the temporal profile of the Integrated Forest Index to identify six land cover change classes: persistent forest, persistent non-forest, persistent water, disturbed forest, post-disturbance forest, and post-disturbance non-forest [48]. Persistent forest and persistent non-forest remained forest or non-forest, respectively, from 1985 to 2006. Disturbed forest was synonymous with forest loss relative to the previous image. Post-disturbance forest and post-disturbance non-forest represented the return to forest or retention of non-forest cover, respectively, following disturbance. By definition, post-disturbance forest and post-disturbance non-forest do not transition to persistent forest or persistent non-forest as the latter two classifications were undisturbed for the entire study period. The accuracy of the forest disturbance maps, assessed for a stratified random sample of pixels by visual comparison of Landsat imagery and high-resolution aerial photography, ranged from 78% to 87% [49].

We quantified the proportion of each land cover class within a 19.7 km radius circle of each BBS route centroid [19], [26]. The 19.7 km buffer radius, in addition to being the shortest buffer radius to encapsulate a BBS route, approximates the median maximum natal dispersal distance of 22.9 km for 150 forest-associated species of North American landbirds (C. Flather, unpublished data) based on allometric relationships developed by Sutherland et al. [50], providing an ecologically relevant scale to link avian nesting and juvenile habitat to the landscape. We included in the analysis only BBS routes for which >80% of the area of this circular landscape was located within a study area (i.e., within the area of a Landsat scene).

### Statistical analyses

Many factors are known to influence BBS counts including environmental effects, species effects, and observer effects. Thus, approaches for analyzing BBS data should be adjusted accordingly. We followed standard protocols for minimizing bias due to environmental effects by removing routes surveyed during inclement weather or outside of the time standards [37]. We minimized species effects by omitting unidentified species and species with <30 route-year observations within the continental United States over the period of the BBS (accidental or low occurrence species), and by summing counts among BBS-tracked subspecies (e.g., red-shafted flicker, yellow-shafted flicker) to the species level (e.g., northern flicker). We also adopted the standard protocol of excluding routes surveyed by novice (first-year) observers [51]. Observer effects also may be introduced with a change in observer on a route over time due to inherent differences in observers' detection abilities [52]. If each observer surveyed only 1 BBS route, the effect of observer would be nested within the route effect. However, a substantial portion of BBS observers conducted surveys on >1 BBS route, causing the grouping factor of observer to lose the nesting property. Thus, we modeled observer and route as separate (crossed) effects.

To assess changes in avian community structure over time, we fit mixed-effects models by guild of richness, abundance, and community similarity measured on BBS routes over time. The mixed-effects model framework allowed random-effects terms that characterized the variation induced in the response by the different observers and routes in addition to fixed-effects terms. The basic structure for the mixed-effects model was:
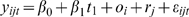
(3)Where 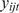 was richness, abundance, or community similarity for the *i*th observer of route *j* at time *t*, 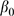 was the common intercept term, 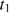 was the variable year and 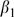 the associated fixed effect of year, which was identical for all groups, 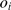 was the random effect for the *i* ^th^ observer, 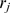 was the random effect for the *j* ^th^ route, and 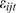 was the error term, assumed to follow a multivariate normal distribution.

To determine the association between forest disturbance and community similarity, we added fixed effects of latitude, longitude, and route attributes of land cover change class (persistent forest, disturbed forest, post-disturbance forest, and post-disturbance non-forest) to equation 3. We fit a global model of all fixed and random effects using a penalized quasi-likelihood estimation method to obtain restricted maximum likelihood estimates of fixed-effects and covariance parameters. We used model selection with AIC to rank models and to determine the most-supported model for each guild. The candidate model set for each guild included models of persistent forest, disturbed forest, post-disturbance forest, post-disturbance non-forest, disturbed forest and post-disturbance non-forest, and disturbed forest with post-disturbance forest. We also included a model containing latitude and longitude, as well as fit all land cover change class models with and without latitude and longitude. The candidate model set contained 14 models, including the global model.

We used a *t*-statistic to approximate variable significance, specifying an upper bound for the degrees of freedom as the number of observations less the number of fixed-effect parameters. This upper bound may be anticonservative at small sample sizes (e.g., <100), meaning that p-values for some variables in final models may be too small because random-effect parameters are not considered when determining degrees of freedom for significance tests. However, the upper bound is generally viewed as acceptable for large sample sizes [53]. We fit all models using the lme4 package within the R language and environment for statistical analysis, version 2.8.1 [54].

## Supporting Information

Table S1 Scientific names of forest bird species and guild classifications by migratory habit and nest location.
(0.38 MB DOC)
