# Supplementary material for: Conservation of Forest Birds: Evidence of a Shifting Baseline in Community Structure
Source: PLoS One. 2010 Aug 2;5(8):e11938. doi: 10.1371/journal.pone.0011938 (PMC2914041; doi:10.1371/journal.pone.0011938)
Supplement: Table S1 — Scientific names of forest bird species and guild classifications by migratory habit and nest location. (0.38 MB DOC) [file pone.0011938.s001.doc]

|  |  | Migratory habit | | |  | Nest location | | | |
| --- | --- | --- | --- | --- | --- | --- | --- | --- | --- |
| Scientific name | Common name | Neotropical migrant | Temperate migrant | Permanent resident |  | Ground | Canopy | Cavity | Forest Interior |
| *Bonasa umbellus* | Ruffed Grouse |  |  | x |  | x |  |  |  |
| *Dendragapus obscurus* and *D. fuliginosus* | Blue Grouse |  |  | x |  | x |  |  |  |
| *Meleagris gallopavo* | Wild Turkey |  |  | x |  | x |  |  |  |
| *Oreortyx pictus* | Mountain Quail |  |  | x |  | x |  |  |  |
| *Elanoides forficatus* | Swallow-Tailed Kite | x |  |  |  |  |  |  |  |
| *Ictinia mississippiensis* | Mississippi Kite | x |  |  |  |  |  |  |  |
| *Accipiter striatus* | Sharp-Shinned Hawk |  | x |  |  |  |  |  |  |
| *Accipiter cooperii* | Cooper's Hawk |  | x |  |  |  |  |  |  |
| *Accipiter gentilis* | Northern Goshawk |  |  | x |  |  |  |  | x |
| *Buteo lineatus* | Red-Shouldered Hawk |  | x |  |  |  |  |  | x |
| *Buteo platypterus* | Broad-Winged Hawk | x |  |  |  |  |  |  | x |
| *Falco sparverius* | American Kestrel |  | x |  |  |  |  | x |  |
| *Patagioenas fasciata* | Band-Tailed Pigeon | x |  |  |  |  |  |  |  |
| *Coccyzus americanus* | Yellow-Billed Cuckoo | x |  |  |  |  | x |  |  |
| *Coccyzus erythropthalmus* | Black-Billed Cuckoo | x |  |  |  |  | x |  |  |
| *Bubo virginianus* | Great Horned Owl |  |  | x |  |  |  |  |  |
| *Glaucidium gnoma* | Northern Pygmy-Owl |  |  | x |  |  |  | x |  |
| *Strix occidentalis* | Spotted Owl |  |  | x |  |  |  |  | x |
| *Strix varia* | Barred Owl |  |  | x |  |  |  | x | x |
| *Asio otus* | Long-Eared Owl |  | x |  |  |  | x | x |  |
| *Aegolius acadicus* | Northern Saw-Whet Owl |  | x |  |  |  |  | x |  |
| *Caprimulgus carolinensis* | Chuck-Will's-Widow | x |  |  |  | x |  |  |  |
| *Caprimulgus vociferus* | Whip-Poor-Will | x |  |  |  | x |  |  |  |
| *Chaetura vauxi* | Vaux's Swift | x |  |  |  |  |  | x |  |
| *Cynanthus latirostris* | Broad-Billed Hummingbird |  | x |  |  |  |  |  |  |
| *Archilochus colubris* | Ruby-Throated Hummingbird | x |  |  |  |  |  |  |  |
| *Archilochus alexandri* | Black-Chinned Hummingbird | x |  |  |  |  |  |  |  |
| *Selasphorus rufus* | Rufous Hummingbird | x |  |  |  |  |  |  |  |
| *Melanerpes erythrocephalus* | Red-Headed Woodpecker |  | x |  |  |  |  | x |  |
| *Melanerpes formicivorus* | Acorn Woodpecker |  |  | x |  |  |  | x |  |
| *Melanerpes carolinus* | Red-Bellied Woodpecker |  |  | x |  |  |  | x |  |
| *Sphyrapicus thyroideus* | Williamson's Sapsucker |  | x |  |  |  |  | x |  |
| *Sphyrapicus varius* | Yellow-Bellied Sapsucker |  | x |  |  |  |  | x |  |
| *Sphyrapicus nuchalis* | Red-Naped Sapsucker |  | x |  |  |  |  | x |  |
| *Sphyrapicus ruber* | Red-Breasted Sapsucker |  | x |  |  |  |  | x |  |
| *Picoides scalaris* | Ladder-Backed Woodpecker |  |  | x |  |  |  | x |  |
| *Picoides nuttallii* | Nuttall's Woodpecker |  |  | x |  |  |  | x |  |
| *Picoides pubescens* | Downy Woodpecker |  |  | x |  |  |  | x |  |
| *Picoides villosus* | Hairy Woodpecker |  |  | x |  |  |  | x |  |
| *Picoides arizonae* | Arizona Woodpecker |  |  | x |  |  |  | x |  |
| *Picoides borealis* | Red-Cockaded Woodpecker |  |  | x |  |  |  | x | x |
| *Picoides albolarvatus* | White-Headed Woodpecker |  |  | x |  |  |  | x | x |
| *Picoides dorsalis* | American Three-Toed Woodpecker |  |  | x |  |  |  | x |  |
| *Picoides arcticus* | Black-Backed Woodpecker |  |  | x |  |  |  | x |  |
| *Dryocopus pileatus* | Pileated Woodpecker |  |  | x |  |  |  | x | x |
| *Contopus cooperi* | Olive-Sided Flycatcher | x |  |  |  |  | x |  | x |
| *Contopus pertinax* | Greater Pewee |  | x |  |  |  |  |  |  |
| *Contopus sordidulus* | Western Wood-Pewee | x |  |  |  |  | x |  |  |
| *Contopus virens* | Eastern Wood-Pewee | x |  |  |  |  | x |  |  |
| *Empidonax flaviventris* | Yellow-Bellied Flycatcher | x |  |  |  | x |  |  |  |
| *Empidonax virescens* | Acadian Flycatcher | x |  |  |  |  | x |  | x |
| *Empidonax minimus* | Least Flycatcher | x |  |  |  |  | x |  |  |
| *Empidonax hammondii* | Hammond's Flycatcher | x |  |  |  |  | x |  | x |
| *Empidonax oberholseri* | Dusky Flycatcher | x |  |  |  |  |  |  |  |
| *Empidonax difficilis* | Pacific-Slope Flycatcher | x |  |  |  | x |  |  |  |
| *Empidonax occidentalis* | Cordilleran Flycatcher | x |  |  |  | x |  |  |  |
| *Pyrocephalus rubinus* | Vermilion Flycatcher | x |  |  |  |  | x |  |  |
| *Myiarchus crinitus* | Great Crested Flycatcher | x |  |  |  |  | x | x |  |
| *Myiarchus tyrannulus* | Brown-Crested Flycatcher |  | x |  |  |  | x | x |  |
| *Tyrannus couchii* | Couch's Kingbird |  | x |  |  |  | x |  |  |
| *Tyrannus vociferans* | Cassin's Kingbird | x |  |  |  |  | x |  |  |
| *Tyrannus verticalis* | Western Kingbird | x |  |  |  |  | x | x |  |
| *Tyrannus dominicensis* | Gray Kingbird |  | x |  |  |  |  |  |  |
| *Vireo griseus* | White-Eyed Vireo | x |  |  |  | x |  |  |  |
| *Vireo flavifrons* | Yellow-Throated Vireo | x |  |  |  |  | x |  |  |
| *Vireo plumbeus* | Plumbeous Vireo | x |  |  |  |  | x |  |  |
| *Vireo cassinii* | Cassin's Vireo | x |  |  |  |  | x |  |  |
| *Vireo solitarius* | Blue-Headed Vireo | x |  |  |  |  | x |  |  |
| *Vireo huttoni* | Hutton's Vireo |  |  | x |  |  | x |  |  |
| *Vireo philadelphicus* | Philadelphia Vireo | x |  |  |  |  | x |  |  |
| *Vireo olivaceus* | Red-Eyed Vireo | x |  |  |  |  | x |  | x |
| *Vireo altiloquus* | Black-Whiskered Vireo |  | x |  |  |  |  |  |  |
| *Perisoreus canadensis* | Gray Jay |  |  | x |  |  | x |  |  |
| *Cyanocitta stelleri* | Steller's Jay |  |  | x |  |  | x |  |  |
| *Cyanocitta cristata* | Blue Jay |  | x |  |  |  | x |  |  |
| *Cyanocorax yncas* | Green Jay |  |  | x |  |  | x |  |  |
| *Aphelocoma coerulescens* | Florida Scrub-Jay |  |  | x |  |  |  |  |  |
| *Aphelocoma californica* | Western Scrub-Jay |  |  | x |  |  |  |  |  |
| *Aphelocoma ultramarina* | Mexican Jay |  |  | x |  |  |  |  |  |
| *Gymnorhinus cyanocephalus* | Pinyon Jay |  |  | x |  |  | x |  |  |
| *Nucifraga columbiana* | Clark's Nutcracker |  |  | x |  |  | x |  |  |
| *Corvus brachyrhynchos* | American Crow |  | x |  |  |  | x |  |  |
| *Corvus caurinus* | Northwestern Crow |  |  | x |  |  | x |  |  |
| *Corvus ossifragus* | Fish Crow |  | x |  |  |  | x |  |  |
| *Corvus corax* | Common Raven |  |  | x |  |  | x |  |  |
| *Poecile carolinensis* | Carolina Chickadee |  |  | x |  |  | x | x |  |
| *Poecile atricapillus* | Black-Capped Chickadee |  |  | x |  |  | x | x |  |
| *Poecile gambeli* | Mountain Chickadee |  |  | x |  |  | x | x |  |
| *Poecile rufescens* | Chestnut-Backed Chickadee |  |  | x |  |  | x | x |  |
| *Poecile hudsonica* | Boreal Chickadee |  |  | x |  |  | x | x |  |
| *Baeolophus wollweberi* | Bridled Titmouse |  |  | x |  |  |  | x |  |
| *Baeolophus inornatus* | Oak Titmouse |  |  | x |  |  | x | x |  |
| *Baeolophus ridgwayi* | Juniper Titmouse |  |  | x |  |  | x | x |  |
| *Baeolophus bicolor* | Tufted Titmouse |  |  | x |  |  | x | x |  |
| *Baeolophus atricristatus* | Black-Crested Titmouse |  |  | x |  |  | x | x |  |
| *Psaltriparus minimus* | Bushtit |  |  | x |  |  | x |  |  |
| *Sitta canadensis* | Red-Breasted Nuthatch |  | x |  |  |  | x | x | x |
| *Sitta carolinensis* | White-Breasted Nuthatch |  |  | x |  |  | x | x | x |
| *Sitta pygmaea* | Pygmy Nuthatch |  |  | x |  |  | x | x |  |
| *Sitta pusilla* | Brown-Headed Nuthatch |  |  | x |  |  | x | x |  |
| *Certhia americana* | Brown Creeper |  | x |  |  |  | x | x | x |
| *Salpinctes obsoletus* | Rock Wren |  | x |  |  | x |  | x |  |
| *Catherpes mexicanus* | Canyon Wren |  |  | x |  | x |  | x |  |
| *Thryothorus ludovicianus* | Carolina Wren |  |  | x |  |  |  | x |  |
| *Thryomanes bewickii* | Bewick's Wren |  | x |  |  |  |  | x |  |
| *Troglodytes aedon* | House Wren | x | x |  |  |  | x | x |  |
| *Troglodytes troglodytes* | Winter Wren |  | x |  |  | x |  | x | x |
| *Cinclus mexicanus* | American Dipper |  |  | x |  | x |  |  |  |
| *Regulus satrapa* | Golden-Crowned Kinglet |  | x |  |  |  | x |  |  |
| *Regulus calendula* | Ruby-Crowned Kinglet |  | x |  |  |  | x |  |  |
| *Polioptila caerulea* | Blue-Gray Gnatcatcher | x |  |  |  |  | x |  |  |
| *Myadestes townsendi* | Townsend's Solitaire |  | x |  |  | x |  |  |  |
| *Catharus fuscescens* | Veery | x |  |  |  | x |  |  |  |
| *Catharus ustulatus* | Swainson's Thrush | x |  |  |  | x |  |  |  |
| *Catharus guttatus* | Hermit Thrush |  | x |  |  | x |  |  | x |
| *Hylocichla mustelina* | Wood Thrush | x |  |  |  |  | x |  | x |
| *Turdus migratorius* | American Robin |  | x |  |  |  | x |  |  |
| *Ixoreus naevius* | Varied Thrush |  | x |  |  |  | x |  | x |
| *Chamaea fasciata* | Wrentit |  |  | x |  |  |  |  |  |
| *Dumetella carolinensis* | Gray Catbird | x |  |  |  |  |  |  |  |
| *Toxostoma rufum* | Brown Thrasher |  | x |  |  |  |  |  |  |
| *Bombycilla cedrorum* | Cedar Waxwing |  | x |  |  |  | x |  |  |
| *Phainopepla nitens* | Phainopepla | x |  |  |  |  | x |  |  |
| *Peucedramus taeniatus* | Olive Warbler |  | x |  |  |  |  |  |  |
| *Vermivora pinus* | Blue-Winged Warbler | x |  |  |  | x |  |  |  |
| *Vermivora chrysoptera* | Golden-Winged Warbler | x |  |  |  | x |  |  |  |
| *Vermivora peregrina* | Tennessee Warbler | x |  |  |  | x |  |  |  |
| *Vermivora celata* | Orange-Crowned Warbler | x |  |  |  | x |  |  |  |
| *Vermivora ruficapilla* | Nashville Warbler | x |  |  |  | x |  |  |  |
| *Vermivora virginiae* | Virginia's Warbler | x |  |  |  | x |  |  |  |
| *Vermivora luciae* | Lucy's Warbler | x |  |  |  |  | x |  |  |
| *Parula americana* | Northern Parula | x |  |  |  |  | x |  |  |
| *Dendroica pensylvanica* | Chestnut-Sided Warbler | x |  |  |  | x |  |  |  |
| *Dendroica magnolia* | Magnolia Warbler | x |  |  |  |  | x |  |  |
| *Dendroica tigrina* | Cape May Warbler | x |  |  |  |  | x |  | x |
| *Dendroica caerulescens* | Black-Throated Blue Warbler | x |  |  |  |  | x |  | x |
| *Dendroica nigrescens* | Black-Throated Gray Warbler | x |  |  |  |  | x |  |  |
| *Dendroica virens* | Black-Throated Green Warbler | x |  |  |  |  | x |  |  |
| *Dendroica townsendi* | Townsend's Warbler | x |  |  |  |  | x |  |  |
| *Dendroica occidentalis* | Hermit Warbler | x |  |  |  |  | x |  |  |
| *Dendroica fusca* | Blackburnian Warbler | x |  |  |  |  | x |  | x |
| *Dendroica dominica* | Yellow-Throated Warbler | x |  |  |  |  | x |  |  |
| *Dendroica graciae* | Grace's Warbler | x |  |  |  |  | x |  |  |
| *Dendroica pinus* | Pine Warbler |  | x |  |  |  | x |  |  |
| *Dendroica discolor* | Prairie Warbler | x |  |  |  |  |  |  |  |
| *Dendroica palmarum* | Palm Warbler | x |  |  |  | x |  |  |  |
| *Dendroica castanea* | Bay-Breasted Warbler | x |  |  |  |  | x |  |  |
| *Dendroica striata* | Blackpoll Warbler | x |  |  |  |  | x |  |  |
| *Dendroica cerulea* | Cerulean Warbler | x |  |  |  |  | x |  | x |
| *Mniotilta varia* | Black-And-White Warbler | x |  |  |  | x |  |  |  |
| *Setophaga ruticilla* | American Redstart | x |  |  |  |  | x |  | x |
| *Protonotaria citrea* | Prothonotary Warbler | x |  |  |  |  | x | x |  |
| *Helmitheros vermivorum* | Worm-Eating Warbler | x |  |  |  | x |  |  | x |
| *Limnothlypis swainsonii* | Swainson's Warbler | x |  |  |  | x |  |  | x |
| *Seiurus aurocapilla* | Ovenbird | x |  |  |  | x |  |  | x |
| *Seiurus noveboracensis* | Northern Waterthrush | x |  |  |  | x |  |  | x |
| *Seiurus motacilla* | Louisiana Waterthrush | x |  |  |  | x |  |  | x |
| *Oporornis formosus* | Kentucky Warbler | x |  |  |  | x |  |  | x |
| *Oporornis agilis* | Connecticut Warbler | x |  |  |  | x |  |  |  |
| *Oporornis philadelphia* | Mourning Warbler | x |  |  |  | x |  |  |  |
| *Oporornis tolmiei* | Macgillivray's Warbler | x |  |  |  | x |  |  |  |
| *Wilsonia citrina* | Hooded Warbler | x |  |  |  |  |  |  | x |
| *Wilsonia pusilla* | Wilson's Warbler | x |  |  |  | x |  |  |  |
| *Wilsonia canadensis* | Canada Warbler | x |  |  |  | x |  |  | x |
| *Cardellina rubrifrons* | Red-Faced Warbler | x |  |  |  | x |  |  |  |
| *Icteria virens* | Yellow-Breasted Chat | x |  |  |  |  |  |  |  |
| *Piranga flava* | Hepatic Tanager | x |  |  |  |  | x |  |  |
| *Piranga rubra* | Summer Tanager | x |  |  |  |  | x |  |  |
| *Piranga olivacea* | Scarlet Tanager | x |  |  |  |  | x |  | x |
| *Piranga ludoviciana* | Western Tanager | x |  |  |  |  | x |  |  |
| *Pipilo maculatus* | Spotted Towhee |  | x |  |  | x |  |  |  |
| *Pipilo erythrophthalmus* | Eastern Towhee |  | x |  |  | x |  |  |  |
| *Aimophila aestivalis* | Bachman's Sparrow |  | x |  |  | x |  |  |  |
| *Spizella atrogularis* | Black-Chinned Sparrow |  | x |  |  |  |  |  |  |
| *Passerella iliaca* | Fox Sparrow |  | x |  |  | x |  |  |  |
| *Melospiza lincolnii* | Lincoln's Sparrow | x |  |  |  |  |  |  |  |
| *Zonotrichia albicollis* | White-Throated Sparrow |  | x |  |  | x |  |  |  |
| *Zonotrichia leucophrys* | White-Crowned Sparrow |  | x |  |  | x |  |  |  |
| *Junco hyemalis* | Dark-Eyed Junco |  |  |  |  | x |  |  |  |
| *Cardinalis cardinalis* | Northern Cardinal |  |  | x |  |  |  |  |  |
| *Pheucticus ludovicianus* | Rose-Breasted Grosbeak | x |  |  |  |  | x |  |  |
| *Pheucticus melanocephalus* | Black-Headed Grosbeak | x |  |  |  |  | x |  |  |
| *Passerina amoena* | Lazuli Bunting | x |  |  |  |  |  |  |  |
| *Passerina cyanea* | Indigo Bunting | x |  |  |  |  |  |  |  |
| *Passerina ciris* | Painted Bunting | x |  |  |  | x |  |  |  |
| *Molothrus ater* | Brown-Headed Cowbird |  | x |  |  |  |  |  |  |
| *Icterus graduacauda* | Audubon's Oriole |  |  | x |  |  |  |  |  |
| *Icterus galbula* | Baltimore Oriole | x |  |  |  |  | x |  |  |
| *Pinicola enucleator* | Pine Grosbeak |  | x |  |  |  | x |  |  |
| *Carpodacus purpureus* | Purple Finch |  | x |  |  |  | x |  |  |
| *Carpodacus cassinii* | Cassin's Finch |  | x |  |  |  | x |  |  |
| *Loxia curvirostra* | Red Crossbill |  | x |  |  |  | x |  |  |
| *Loxia leucoptera* | White-Winged Crossbill |  | x |  |  |  | x |  |  |
| *Carduelis pinus* | Pine Siskin |  | x |  |  |  | x |  |  |
| *Carduelis lawrencei* | Lawrence's Goldfinch |  |  | x |  |  | x |  |  |
| *Coccothraustes vespertinus* | Evening Grosbeak |  | x |  |  |  | x |  |  |
